# Accurate detection of cerebellar smooth pursuit eye movement abnormalities via mobile phone video and machine learning

**DOI:** 10.1038/s41598-020-75661-x

**Published:** 2020-10-29

**Authors:** Zhuoqing Chang, Ziyu Chen, Christopher D. Stephen, Jeremy D. Schmahmann, Hau-Tieng Wu, Guillermo Sapiro, Anoopum S. Gupta

**Affiliations:** 1 Department of Electrical and Computer Engineering, Duke University, Durham, NC USA; 2 Department of Mathematics, Duke University, Durham, NC USA; 3 Ataxia Center and Department of Neurology, Massachusetts General Hospital, Harvard Medical School, 100 Cambridge St, Boston, MA USA; 4 Department of Statistical Science, Duke University, Durham, NC USA; 5 Department of Computer Science and Department of Biomedical Engineering, Duke University, Durham, NC USA

**Keywords:** Spinocerebellar ataxia, Diagnostic markers

## Abstract

Eye movements are disrupted in many neurodegenerative diseases and are frequent and early features in conditions affecting the cerebellum. Characterizing eye movements is important for diagnosis and may be useful for tracking disease progression and response to therapies. Assessments are limited as they require an in-person evaluation by a neurology subspecialist or specialized and expensive equipment. We tested the hypothesis that important eye movement abnormalities in cerebellar disorders (i.e., ataxias) could be captured from iPhone video. Videos of the face were collected from individuals with ataxia (n = 102) and from a comparative population (Parkinson’s disease or healthy participants, n = 61). Computer vision algorithms were used to track the position of the eye which was transformed into high temporal resolution spectral features. Machine learning models trained on eye movement features were able to identify abnormalities in smooth pursuit (a key eye behavior) and accurately distinguish individuals with abnormal pursuit from controls (sensitivity = 0.84, specificity = 0.77). A novel machine learning approach generated severity estimates that correlated well with the clinician scores. We demonstrate the feasibility of capturing eye movement information using an inexpensive and widely accessible technology. This may be a useful approach for disease screening and for measuring severity in clinical trials.

## Introduction

The spinocerebellar ataxias (SCAs) are rare autosomal dominant, typically adult-onset progressive neurologic disorders with a prevalence of between 1–3 per 100,000^1^. These diseases progress slowly over decades^2^ and profoundly affect quality of life^3^. SCAs are characterized by dysfunction of the cerebellum, resulting in unsteady gait, clumsy arm and leg movements, slurred speech, and abnormal eye movements^1^. There are now a number of disease modifying drug development programs aimed at slowing or stopping the progression of SCAs^4–6^. Sensitively identifying the clinical onset of disease and precisely measuring disease severity over time remain important challenges to support drug development efforts and clinical practice.

Eye movement or “oculomotor” abnormalities, including nystagmus (repetitive, uncontrolled eye movements), overshoot, undershoot, and slowed saccades, and abnormalities in smooth pursuit (slow movements used to track objects in motion), are frequent and early features in SCA^7–11^ and progress with disease stage^12^. Abnormalities in smooth pursuit (i.e., saccadic pursuit) in particular were found to be the most prevalent oculomotor sign in SCAs and were often present in early stages of disease^11^. The most common approach for characterizing oculomotor abnormalities in ataxia is through visual examination of eye movements during clinician-administered eye movement tasks. However, eye movement characteristics including saccadic pursuit are difficult to quantify visually, even by ataxia experts, resulting in clinical rating scales that either exclude oculomotor severity scoring (Scale for the Assessment and Rating of Ataxia^13^) or characterize them broadly, for example by evaluating the presence or absence of four cardinal signs as in the Brief Ataxia Rating Scale (BARS)^14^. This important clinical assessment thus has limitations in both precision and objectivity and depends on the experience of the examiner. Given these limitations, it remains unclear if current clinical assessments of oculomotor function can measure disease progression in the context of clinical trials. It is also unclear if clinician-performed assessments are practical or sensitive for following presymptomatic carriers to identify clinical onset.

Research-grade eye tracking using expensive instrumentation in the laboratory provides a means of identifying various neurological disorders^15,16^ including accurately and precisely quantifying individual oculomotor abnormalities in ataxias^17^. However, this is only available in specialized centers and are even less accessible in rural areas and to disease populations where mobility is affected^18^. Furthermore, frequent and longitudinal assessments for tracking disease severity in a clinical trial and interval monitoring of presymptomatic gene carriers to identify clinical onset may be less practical using these technologies.

To address the limited precision and objectivity of clinician-performed oculomotor assessments and the accessibility and scalability limitations of laboratory performed assessments, sensitive and widely available instruments for assessing oculomotor function are needed. Such tools may be useful for detecting early signs of ataxia, for identifying onset of clinical disease in SCAs, and for measuring disease progression over time. Similar instruments are needed broadly for neurodegenerative disorders which cause eye movement abnormalities, including movement disorders (Huntington’s disease, progressive supranuclear palsy, multiple system atrophy) and dementias (frontotemporal dementia, Alzheimer’s disease)^19^.

We have developed a scalable and inexpensive system for quantifying abnormalities in smooth pursuit in individuals with ataxia using a mobile device camera to record eye movements while viewing stimuli on a tablet screen. We demonstrate that this system combined with signal processing and machine learning techniques, can accurately and rapidly detect abnormalities in smooth pursuit and grade the severity of oculomotor dysfunction in cerebellar ataxias.

## Methods

### Collection of data

#### Standard protocol approvals, registrations, and patient consents

All experiment protocols were approved by the Partners Healthcare Institutional Review Board and are in accordance with guidelines of the Declaration of Helsinki. All participants provided written informed consent to participate in the study.

#### Participant selection

Participants were recruited from the Massachusetts General Hospital (MGH) between September 2017 and January 2019 from the Ataxia and Movement Disorders Units. Additionally, children with ataxia-telangiectasia (A-T) were recruited through the Ataxia-Telangiectasia Children’s Project or the MGH Ataxia Unit. Individuals were invited (but not required) to repeat a testing session at a subsequent visit to MGH. Healthy control data were obtained from two populations: (1) family members of patients (e.g., asymptomatic partners or gene negative family members); and (2) MGH staff. Clinical data for MGH patients including disease diagnosis and scores on clinical rating scales were identified in the medical record from their concurrent visit. All patients had disease-specific rating scale scores and for those without a same-day clinical appointment, scores were obtained from video data of the same-day neurological exam after review by A.S.G., a movement disorders and ataxia specialist.

#### Participant demographics

**Figure 1:**
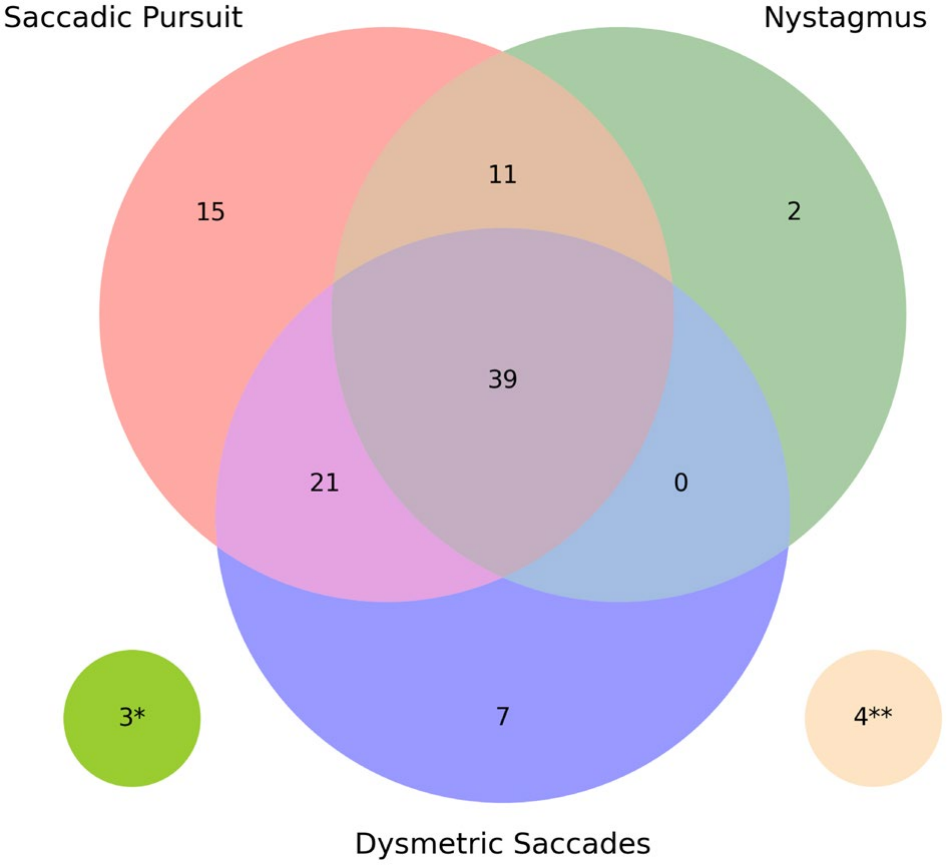
Oculomotor abnormality distribution of ataxia participants. (**) 4 participants do not have any oculomotor abnormalities. (*) 3 participants do not have saccadic pursuit, nystagmus, or dysmetric saccades (but have gaze holding abnormalities and/or slowed saccades). The information pertaining to the presence or absence of these oculomotor signs were extracted from the clinical medical record.

We collected video data on 201 ataxia, Parkinson’s disease, and control participants in the clinic setting. Data from 10 ataxia participants were excluded due to incomplete clinical documentation of oculomotor features. We also excluded data from 14 participants who intermittently directed their gaze away from the stimuli and/or moved their head in excess (i.e., did not perform the task as directed). In addition, 11 participants were excluded due to inability of the face detection algorithm to detect the subject’s face in large portions of the video; 2 participants’ data were excluded due to technical issues with data collection that resulted in incorrect video frame rate capture; and 1 participant was excluded due to excessive blinking. Of the remaining 163 participants (99 male, 64 female) whose data we used for analysis, 102 had cerebellar ataxia, 43 had Parkinson’s disease, and 18 were healthy controls. 95 of the 102 ataxia patients had one or more abnormalities on clinical oculomotor assessment. All Parkinson’s disease patients had normal oculomotor function according to chart review. It is well known that individuals with Parkinson’s disease can have saccadic pursuit and other oculomotor abnormalities^20^ (although less severe than in cerebellar ataxias). It is therefore possible that subtle findings were missed on the clinical assessment, which would result in an underestimation of performance for our classification models. The demographics of the 163 participants are shown in Table 1. The disease composition of the 102 ataxia participants are shown in Fig. 1.

**Table 1:** Participants demographics.

	Clinical		
	Controls	Ataxia	Parkinson’s disease
N	18	102 (total) 31 with SCA (11 SCA-3, 6 SCA-1, 6 SCA-6, and 3 SCA-2), 13 A-T, 8 MSA-C, 3 FA, 2 ARCA-1, 2 SPG-7	43
Age	3–37 (M = 18.1, SD = 10.5)	7–78 (M = 53.0, SD = 19.2)	45–82 (M = 67.0, SD = 8.0)
Sex	66.7% male, 33.3% female	54.9% male, 45.1% female	72.1% male, 27.9% female
Oculomotor severity (clinical score on BARS or UPDRS)		BARS (scale 0–2): 0–2 (M = 1.1, SD = 0.6)	
Disease severity (overall clinical score on BARS or UPDRS)		BARS (scale 0–30): 1–23.5 (M = 10.7, SD = 5.6)	UPDRS Part III (scale 0–108): 1–35 (M = 13.9, SD = 6.8)

*M* mean, *SD* standard deviation, *UPDRS* Unified Parkinson’s disease Rating Scale, *BARS* Brief Ataxia Rating Scale, *SCA* spinocerebellar ataxia, *A-T* ataxia-telangiectasia, *MSA-C* multiple system atrophy, cerebellar-type, *FA* Friedreich's ataxia, *ARCA-1* autosomal recessive cerebellar ataxia type 1, *SPG-7* spastic paraplegia type 7.

#### Clinical data collection procedures

All neurologic examinations were videotaped. Ataxia patients were scored on the Brief Ataxia Rating Scale (BARS^14^) (range 0–30) by movement disorders and ataxia specialists (C.D.S., J.D.S., and A.S.G.), which includes a score for oculomotor function (range 0–2). The BARS oculomotor score is generated by adding a half point for the presence of each of four cardinal oculomotor signs: eye movements present in primary position (i.e., at rest), abnormalities in smooth pursuit (i.e., saccadic pursuit), hypometric (catch-up/undershoot) and/or hypermetric (overshoot) saccades, and gaze-evoked nystagmus. In addition to utilizing the aggregate oculomotor score for analysis, we obtained information about the presence or absence of each cardinal sign for each individual from the medical record. Individuals with a diagnosis of idiopathic Parkinson’s disease were assumed to not have any of the four cardinal ataxia oculomotor signs as defined by BARS unless otherwise noted in the movement disorders specialist clinical note. This population was therefore used as a control population for comparison with ataxia. As described above, this assumption could result in an underestimation of classification model performance. Throughout the paper, there is reference to the “Typical” group; this term refers to the group of participants without clinical oculomotor abnormalities (i.e., healthy controls and Parkinson’s disease participants and 4 ataxia participants with a BARS oculomotor score of zero, N = 65).

#### Video oculomotor data collection procedures

**Figure 2:**
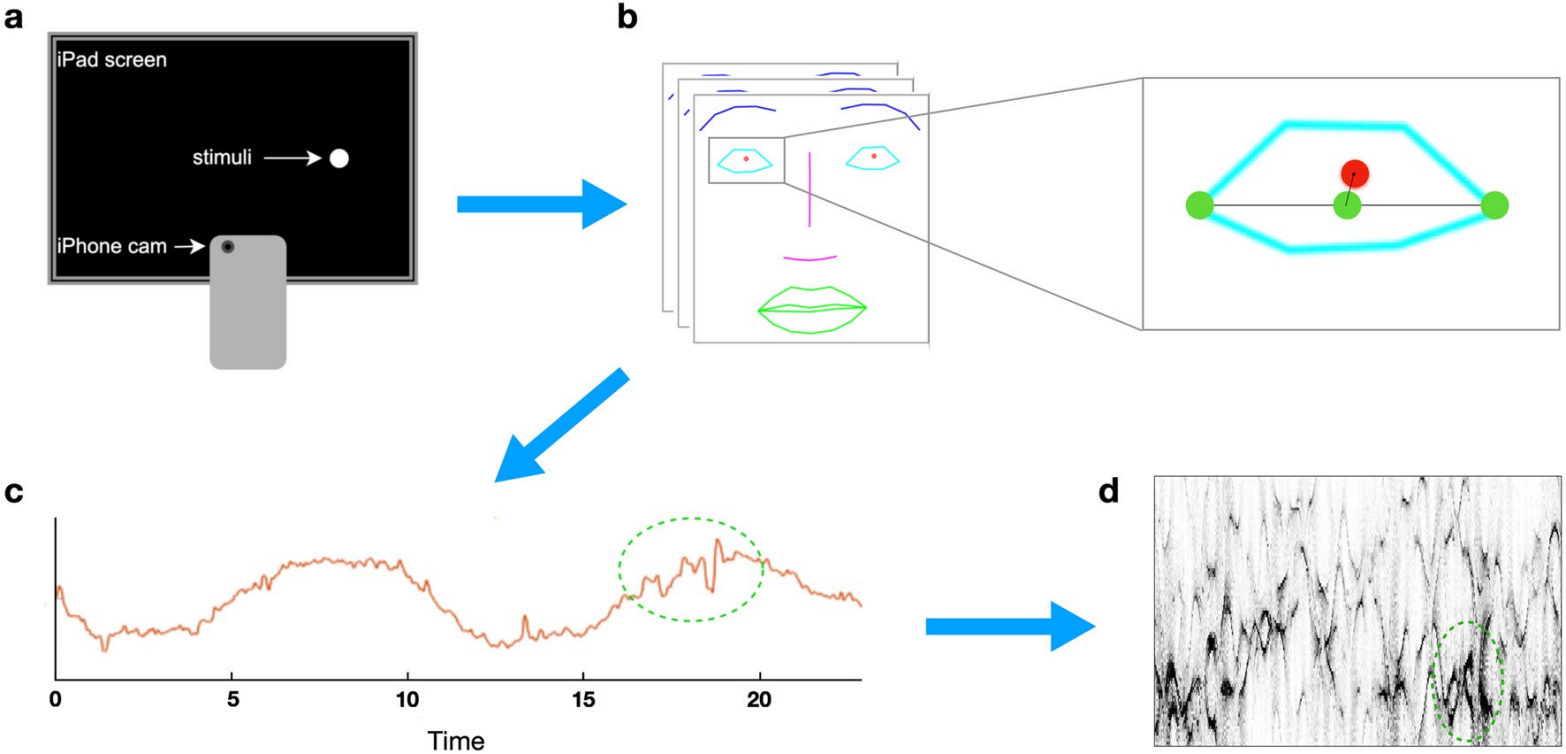
Flow diagram of data processing and feature extraction steps. (**a**) iPad-iPhone configuration. The iPhone captures a slow-motion video (240 frames per second at 1080p) of the participant's face while they are following a moving dot on the iPad screen with their eyes. Each cycle of the stimuli (center to both sides of the screen and back) lasted 2 min. (**b**) Facial landmarks are extracted from the video frames using Intraface^22^. The normalized iris center is computed using the midpoint of the eye corner landmarks as the origin. (**c**) The X (horizontal) coordinate of the normalized iris center is collected across frames to obtain the normalized iris trajectory. Abnormal eye movement from an ataxia patient is highlighted in green. (**d**) Time–frequency information is obtained using ConceFT^25^. Horizontal axis represents time and vertical axis represents frequency; darker regions indicate stronger signals. The green circle highlights the quantitative frequency information corresponding to the abnormal eye movement.

Participants were seated approximately one foot in front of an iPad Pro 12.9-inch (2nd gen) and iPhone 8+ configuration (both, Apple, Cupertino, CA) illustrated in Fig. 2a. A custom iOS application on the iPad led participants through a smooth pursuit task paradigm that was composed of 2 trials. The parameters of the trials (i.e., speed and amplitude) were designed to match closely with the clinical oculomotor examination of smooth pursuit, in which a clinician asks the subject to follow their finger as it moves with as constant as possible velocity across the visual field a few times, as well as with smooth pursuit paradigms used in prior work in SCAs^21^. During each trial, a dot would appear at the center of the screen and move horizontally for 2 cycles (a cycle is defined as the dot starting from the center to returning to the center after reaching the 2 extremities). The dot moved continuously throughout each trial only stopping for 2.5 s at the extremities of the horizontal trajectory (16-degree amplitude). The dot moved at approximately 11 degrees per second during the first trial (T1) and approximately 16 degrees per second during the second trial (T2). Two different speeds were used to account for variability in how different clinicians may perform the task. The entire task was less than 2 min long. While performing the task, the participant’s face was recorded using the rear camera of the iPhone at either 720p × 240 frames per second (fps) or 1080p × 240 fps. No chin rest was used during the data collection process, but participants were instructed to keep their head as still as possible.

### Data processing

#### Processing of movement data and feature extraction

**Figure 3:**
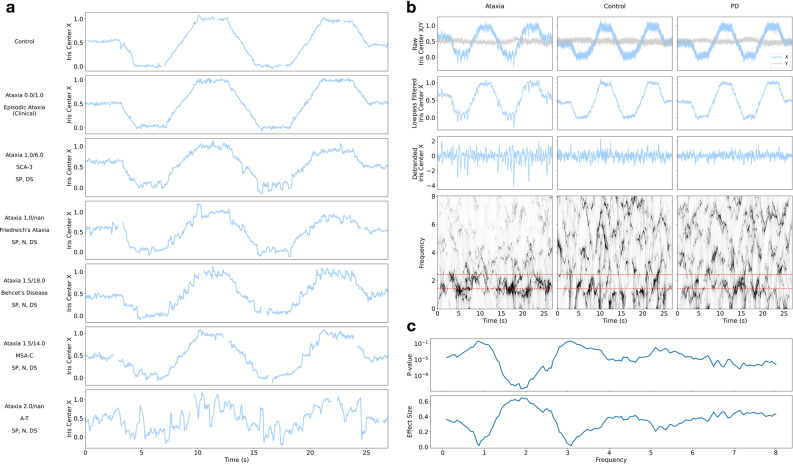
Normalized iris trajectory and ConceFT plot. (**a**) Examples of normalized iris X coordinate trajectory for participants with different diagnoses, ataxia severity, and oculomotor abnormality. The participant's general diagnosis (ataxia or control), BARS oculomotor score and BARS total score (shown as X/Y), specific ataxia type, and presence of oculomotor abnormalities (*SP* saccadic pursuit, *N* nystagmus, *DS *dysmetric saccades) is shown on left side. Missing data in the signal is due to either undetected facial landmarks or filtering of blinks. “Nan” in place of BARS total score was listed when total BARS score was not available. *SCA* spinocerebellar ataxia, *A-T* ataxia-telangiectasia, *MSA-C* multiple system atrophy, cerebellar-type. (**b**) Examples of normalized, low pass filtered, and detrended iris X (and Y) coordinate trajectories and their corresponding ConceFT plot for different diagnosis groups. Darker regions in the ConceFT plot indicate relatively stronger signal power. Ataxia patients display more power in the 1.5–2.5 Hz frequency band (the region between the red dotted lines). (**c**) P value and effect size of relative band power at different frequencies between participants with saccadic pursuit and participants without any oculomotor abnormalities. P values are computed using the standard Mann–Whitney *U* test and effect size is measured using the also standard rank-biserial correlation.

We used Intraface^22^ to detect 12 eye and 2 iris center facial landmarks for each frame in a participant's video. To account for head movement, we compute, for each eye, the normalized iris center (NIC) as the iris center position relative to the midpoint of the 2 eye corner landmarks (Fig. 2b). We use the X (horizontal) coordinate of the NIC corresponding to frames when the dot moved horizontally (Fig. 2c) in subsequent processing steps due to it having much higher signal to noise ratio compared to the Y (vertical) coordinate (an example of the X and Y coordinates are shown in Fig. 3b). Examples of the normalized iris trajectory are shown in Fig. 3a. All analyses were performed on the left eye position signal since there are no substantial left–right asymmetries in the neurodegenerative ataxias.

Eye blinks appear as sharp peaks in the normalized iris signal due to sudden position changes of the eye landmarks which severely affects the extraction of spectral features in later stages. We detect blink frames using the eye aspect ratio^23^. The NIC corresponding to blink frames are then recomputed using cubic interpolation for the subsequent spectral transformation. The spectral power for these interpolated frames is ignored in the final feature computation.

A low-pass filter with a cutoff frequency of 8 Hz is used to first denoise the blink-filtered interpolated signal. We then use a repeated median filter^24^ for detrending before applying ConceFT^25^, a novel method to determine the time–frequency content of time-dependent signals consisting of multiple oscillatory components with time-varying amplitudes and instantaneous frequencies, to acquire a time–frequency representation of the signal (Fig. 2d). Examples of the raw, low pass filtered, and detrended NIC signal and its corresponding ConceFT representation are shown in Fig. 3b. We divide the 1–8 Hz frequency band into 14 equal segments (1–1.5 Hz, 1.5–2 Hz, …, 7.5–8 Hz) and compute the sum and variance of power within each segment. The sum and variance for each segment are normalized by the total sum and total variance across all segments respectively to derive 14 ConceFT-Sum (normalized sum) and 14 ConceFT-Var (normalized variance) features. We regard them together as the 28 ConceFT features. The 28 ConceFT features were computed for the video segment corresponding to trial 1 (T1) and trial 2 (T2) independently. An additional feature set of 28 features was obtained by averaging the T1 and T2 features.

#### Classification models

Each feature set was standardized to have zero mean and unit standard deviation. For each feature set, leave-one-out cross validation was used to train and test a binary SVM with linear kernel to differentiate between individuals with abnormal smooth pursuit (saccadic pursuit) and individuals with no oculomotor abnormalities. The class weight was set inversely proportional to the number of samples in each class to handle the issue of class imbalance.

#### Score estimation model

A score estimation algorithm was developed that could be more robust to imprecise clinical labels. The algorithm involved two steps. In the first step we performed a pairwise comparison of all participants in the dataset (i.e., individual 1 was pairwise compared with individuals 2 through *N*, and so on). We trained a logistic regression classification model (with L1 regularization) to identify the individual in all pairs with more severe disease. The input to the model was (1) the numerical difference in the 28 ConceFT features between two individuals (e.g., individual 1 minus individual 2); and (2) the binary variable indicating which individual’s oculomotor score on BARS was higher. If all pairwise comparisons between participants were considered, there would be $N*(N - 1)/2$ unique comparisons. However, comparisons between individuals with the same score were excluded. Furthermore, a separate model was trained for each individual (with that individual’s data excluded as in cross-validation). This ensured that in the severity estimation step (second step described below), the estimation was blind to any data from that individual. This process, which was used in prior work to sensitively detect disease progression^26^, enables the model to explicitly learn feature weights that could predict differences in clinical severity. This is important because in other behavioral domains such as arm movement, features informative of ataxia disease severity differ from features informative for distinguishing ataxia from controls^26^.

In the second step, we applied the classification model weights in the first step to the original 28 ConceFT features for each individual to generate the estimated severity score. As described above, models were trained using cross-validation, thus the model weights applied to each individual were blind to that individual’s data. An analogous pairwise comparison approach has been previously used to generate clinical severity estimates in Parkinson’s disease^27^.

## Results

### Frequency content analysis

**Figure 4:**
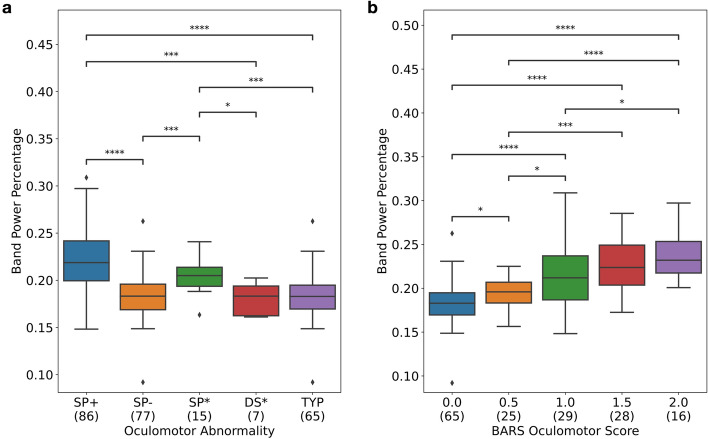
Boxplot of percentage of power in the 1.5–2.5 Hz band for different oculomotor abnormality groups (**a**), different BARS oculomotor score groups (**b**). ‘+', ‘−', and ‘*' indicates the presence, absence, and only presence of an oculomotor abnormality respectively. Typical refers to the group of participants without clinical oculomotor abnormalities (i.e., healthy controls and Parkinson’s disease participants and 4 ataxia patients with a BARS oculomotor score of zero, N = 65). Note that the groups in (**a**) are not mutually exclusive. For each box, the middle line indicates the median, and the bottom and top edges of the box indicate the 25th and 75th percentiles, respectively. The whiskers extend to the most extreme data points not considered outliers. Data points are considered outliers, indicated as a black diamond, if they fall outside approximately 2.7σ, where σ is the standard deviation. * indicates statistical significance with *p* < 0.05, ** indicates statistical significance with *p* < 0.01, *** indicates statistical significance with *p* < 0.001, **** indicates statistical significance *p* < 0.0001. The number of individuals in each group are indicated in parentheses below the group label. *SP* saccadic pursuit, *DS* dysmetric saccades, *TYP* no oculomotor abnormality, “+” presence of the abnormality, “−” absence of the abnormality, “*” presence of only this abnormality (but not other abnormalities).

Visual inspection of eye tracking time series data suggested differences between controls and individuals with ataxia that could be reflected in the frequency content of the signals (Fig. 3a). Relative power across the frequency range of 0.1–8 Hz was computed with ConceFT (example outputs of the algorithm are shown in Fig. 3b). The power spectrum was compared in 0.05 Hz increments for individuals with and without abnormal smooth pursuit (i.e., saccadic pursuit), and demonstrated large differences between the two groups in the 1.5–2.5 Hz range (Fig. 3c). Based on this observation, relative power was aggregated in the 1.5–2.5 Hz range to generate a value that represented the proportion of power in the 1.5–2.5 Hz frequency band. A boxplot of this feature is shown for different eye movement disorder groups (Fig. 4a) and different BARS oculomotor score groups (Fig. 4b). As shown in Fig. 4a, individuals who had abnormalities in smooth pursuit (SP+, N = 86) had significantly higher relative power in the 1.5–2.5 Hz band compared to individuals with no oculomotor abnormalities (Typical, N = 65, *p* < 1 × 10^–11^, effect size = 0.66) and compared to individuals without abnormalities in smooth pursuit but potentially other oculomotor abnormalities (SP-, N = 77, *p* < 1 × 10^–12^, effect size = 0.67). Individuals who only had abnormalities in smooth pursuit and no other oculomotor signs (SP*, N = 15) also had significantly higher power in this band compared to the Typical group (*p* < 0.001, effect size = 0.57). Individuals with dysmetric saccades only and no other oculomotor signs (DS*, N = 7) were not significantly different from the Typical group (*p* > 0.5, effect size = 0.11). There were not enough individuals with nystagmus only (N = 2) for comparison. Overall, these comparisons demonstrate that increased power in the 1.5–2.5 Hz range on this task is relatively specific for abnormalities in smooth pursuit and strongly distinguishes individuals with abnormalities from the Typical group. Selecting frequency ranges of 1.5–3 Hz and 1–3 Hz demonstrated the same statistically significant comparisons (data not shown). Furthermore, power in the 1.5–2.5 Hz band increased with BARS oculomotor severity and demonstrated significant differences between some group pairs with only a half point difference in BARS score (Fig. 4b). The results shown are computed using the averaged features from trial 1 and trial 2. However, similar results were observed using features from either trial independently.

### Classification analyses

Table 2 shows the performance of linear SVM models trained on 28 ConceFT features to distinguish participants with saccadic pursuit (N = 86) from the Typical group (N = 65). Compared to using trial 1 features (T1) and trial 2 features (T2) only, using the combined features (T1 + T2) yielded the best performance with an area under the ROC curve (AUC) of 0.85 and high sensitivity (0.84) and specificity (0.77). When distinguishing ataxia participants from controls and individuals with Parkinson’s, the AUC was 0.72 with sensitivity 0.78 and specificity 0.53. When replacing the linear SVM classifier with linear discriminant analysis and using only 4 features in the 1.5–2.5 Hz band, good performance was also observed for distinguishing participants with saccadic pursuit from individuals without oculomotor abnormalities (AUC = 0.72, sensitivity = 0.74, specificity = 0.72).

**Table 2:** Oculomotor abnormality classification results.

Trial	Classification groups: SP + (86) vs TYP (65)		
	T1	T2	T1 + T2
Area under curve (AUC)	0.72	0.73	0.85
Optimal point sensitivity/specificity	0.69/0.62	0.78/0.54	0.84/0.77
80% True positive point Sensitivity/Specificity	0.80/0.39	0.80/0.48	0.80/0.80
20% False positive point Sensitivity/Specificity	0.54/0.82	0.47/0.82	0.78/0.82

*SP+* with saccadic pursuit abnormality, *TYP* no oculomotor abnormality, *T1* using features from trial 1, *T2* using features from trial 2, *T1 + T2* using averaged features from trial 1 and 2.

### Clinical score estimation

**Figure 5:**
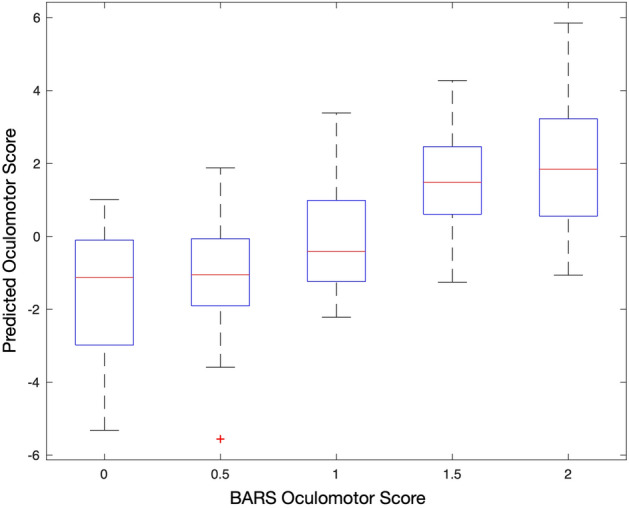
Oculomotor score estimation results. The Pearson correlation coefficient between the predicted oculomotor score and the clinical oculomotor score is 0.63. The Y-axis range is different from the X-axis range due to the type of score estimation model used.

Next we tested whether the spectral content of eye tracking data on the smooth pursuit task, represented by the 28 ConceFT features, contained information about the overall severity of eye movement abnormalities in individuals with ataxia. The BARS oculomotor subscore is nonlinear and composed of information beyond what is assessed on the smooth pursuit task (e.g., abnormalities in primary position gaze holding and saccadic dysmetria). Thus, the purpose of training the model wasn’t to try and achieve high estimation accuracy, but instead to determine if a combination of spectral information correlated with oculomotor severity as measured on BARS. We trained a machine learning model based on pairwise comparisons of individuals (see “Methods”) to estimate the BARS oculomotor subscore with performance evaluated using cross-validation. The Pearson correlation coefficient between the model estimated score and the clinical score was 0.63. A boxplot of the estimated scores is shown in Fig. 5.

## Discussion

We demonstrated that it is feasible to extract iris position data from consumer-grade device video recordings during the performance of standard oculomotor tasks such as smooth pursuit. We were able to extract features from the iris position data that were informative for detecting abnormalities in smooth pursuit and which correlated well with the severity of oculomotor dysfunction. In particular, individuals with abnormal smooth pursuit had increased power in the 1.5–2.5 Hz range, likely reflecting the periodicity of consecutive small saccades performed in order to track a moving target. Additionally, we achieved high sensitivity and specificity for distinguishing individuals with saccadic pursuit from individuals without oculomotor abnormalities based on the spectral content of their eye. Moderate classification results were obtained when classifying individuals with ataxia (even when including individuals without abnormalities in smooth pursuit) from the control group. Finally, our oculomotor severity estimation model demonstrated good correlation with the BARS oculomotor score. Although the information provided to the model and the information provided to the clinician performing the BARS oculomotor score are different, this result indicates that the presented approach for capturing smooth pursuit information may be useful for rating the severity of oculomotor dysfunction in ataxias.

With the acceleration of promising therapy development efforts for cerebellar ataxias, there is a need for tools to improve how we screen and diagnose individuals with ataxia, this being an example of neurodegenerative disorders. Furthermore, for presymptomatic gene-positive individuals, we need technologies that can monitor for clinical onset of disease to help determine when to initiate expensive and potentially invasive therapies. Cerebellar ataxias, like other neurodegenerative diseases, are challenging because of heterogeneity in phenotype, with individual differences in the pattern of the major motor domains affected (speech, eye movement, limb motor control, and gait/balance) as well as in how clinical phenotype manifests and progresses. This heterogeneity underscores the need to develop scalable tools that can assess each of the key motor domains, including eye movement as here addressed. There are efforts to develop tools for speech^28^, gait, and limb^26,29^ assessments using microphone recordings of voice, wearable sensors, and computer input devices. In this work we report a scalable approach for capturing abnormalities in smooth pursuit, an early and characteristic sign in cerebellar ataxias^11^ as well as a sign in other neurodegenerative disorders^19^. We also demonstrate that features of smooth pursuit are correlated with overall oculomotor severity, raising the possibility that this mobile tool could be used to track severity of oculomotor abnormalities over time in natural history studies and clinical trials. We see the use of mobile phone-based video oculomotor assessments as a promising component of a multidomain screening tool for cerebellar ataxias and potentially other neurodegenerative diseases.

With the increasing adoption of accessible and inexpensive devices, such as smartphones, tablets, and webcams, there is potential to move screening and possibly initial diagnosis of neurodegenerative disorders beyond the clinic setting to underserved populations or remote areas. Smartphone applications such as Autism and Beyond^30^ for autism spectrum disorder and MPower^31^ for Parkinson’s disease have already demonstrated the potential for collecting clinically relevant information remotely. These approaches have the potential to reduce the burden on clinicians which is a serious problem in neurodevelopmental and neurodegenerative disorders where there are not enough experts and diagnosis can be challenging and time consuming. In addition to supporting clinical efforts, scalable approaches for oculomotor assessments facilitates new research directions and has the potential to enable an understanding of possible diurnal and/or daily fluctuations in oculomotor function. While there are limitations and potential pitfalls of digital phenotyping, when used correctly it can serve as a means to addressing healthcare challenges and research questions that require widespread use and adoption.

There are several limitations of this study. First, although the iPhone-based iris position data closely reflects the task and expectedly changes as a function of disease class and severity (Fig. 3a), we do not have ground truth for iris position or head position to complement the already validated, though in other applications, computer vision algorithm here employed. Future work simultaneously collecting iPhone video and research-grade eye and face landmark tracking will be important to estimate eye tracking accuracy and contributions from head motion. Second, we do not have ground truth for the severity of smooth pursuit abnormalities, just the presence or absence of abnormalities in smooth pursuit. We will address this in future work by following oculomotor function in individuals with neurodegenerative ataxia diagnoses over time along with clinician grading of the severity of the smooth pursuit abnormalities during their oculomotor assessments. We expect that the graded clinical assessments may enable improved performance of the machine learning models reported here. Third, for future utility as a component of an ataxia screening tool it will be important to train classification models on larger datasets and evaluate performance in a test set with a large proportion of healthy controls, thereby reflecting the true population. The potential scalability of the eye tracking approach allows for the necessary large-scale data collection. Fourth, participants whose data were not usable due to a large amount of head motion or blinking were excluded from the analysis. Providing participants with feedback via real time analysis and developing additional robustness in the computer vision algorithms for iris tracking could potentially address these issues. Fifth, two different devices were used for this study, one for stimulus presentation and one for video recording. Now that there is a fast-speed front facing camera on the most recent iPhone (not just a fast-speed back facing camera), it may be possible to collect the same data on a single device, which would further increase the scalability of the eye tracking approach. Lastly, while the features were computed with a state-of-the-art time–frequency analysis algorithm handling the presence of multiple spontaneous periodic signals and high noise, we can still observe some residual noise and trend information; addressing this, for example with machine learning tools once more data is collected, is likely to improve performance further.

## Data Availability

The data that support the findings of this study are available from the corresponding author upon reasonable request.
